# Whole-genome resequencing of Chinese pangolins reveals a population structure and provides insights into their conservation

**DOI:** 10.1038/s42003-022-03757-3

**Published:** 2022-08-25

**Authors:** Qing Wang, Tianming Lan, Haimeng Li, Sunil Kumar Sahu, Minhui Shi, Yixin Zhu, Lei Han, Shangchen Yang, Qian Li, Le Zhang, Zhangwen Deng, Huan Liu, Yan Hua

**Affiliations:** 1 College of Life Sciences, University of Chinese Academy of Sciences, Beijing, China; 2 State Key Laboratory of Agricultural Genomics, BGI-Shenzhen, Shenzhen, China; 3 BGI Life Science Joint Research Center, Northeast Forestry University, Harbin, China; 4 Guangdong Provincial Key Laboratory of Genome Read and Write, BGI-Shenzhen, Shenzhen, China; 5 College of Wildlife and Protected Area, Northeast Forestry University, Harbin, China; 6 College of Life Sciences, Zhejiang University, Hangzhou, China; 7 Guangxi Forest Inventory and Planning Institute, Nanning, China; 8 Guangdong Provincial Key Laboratory of Silviculture, Protection and Utilization, Guangdong Academy of Forestry, Guangzhou, China

**Keywords:** Conservation genomics, Structural variation, Genetic databases

## Abstract

Poaching and trafficking have a substantial negative impact on the population growth and range expansion of the Chinese pangolin (*Manis pentadactyla*). However, recently reported activities of Chinese pangolins in several sites of Guangdong province in China indicate a promising sign for the recovery of this threatened species. Here, we re-sequence genomes of 15 individuals and perform comprehensive population genomics analyses with previously published 22 individuals. These Chinese pangolins are found to be divided into three distinct populations. Multiple lines of evidence indicate the existence of a newly discovered population (CPA) comprises entirely of individuals from Guangdong province. The other two populations (CPB and CPC) have previously been documented. The genetic differentiation of the CPA and CPC is extremely large (*F*_ST_ = 0.541), which is larger than many subspecies-level differentiations. Even for the closer CPA and CPB, their differentiation (*F*_ST_ = 0.101) is still comparable with the population-level differentiation of many endangered species. Further analysis reveals that the CPA and CPB populations separate 2.5–4.0 thousand years ago (kya), and on the other hand, CPA and CPC diverge around 25–40 kya. The CPA population harbors more runs of homozygosity (ROHs) than the CPB and CPC populations, indicating that inbreeding is more prevalent in the CPA population. Although the CPC population has less mutational load than CPA and CPB populations, we predict that several Loss of Function (LoF) mutations will be translocated into the CPA or CPB populations by using the CPC as a donor population for genetic rescue. Our findings imply that the conservation of Chinese pangolins is challenging, and implementing genetic rescue among the three groups should be done with extreme caution.

## Abstract (web summary)

Whole-genome resequencing of Chinese pangolins reveals a new population CPA that is genetically distinct from and harbor more homozygosity than CPB and CPC populations, indicating prevalence in inbreeding and implying challenges in conservation.

## Introduction

Small populations particularly tend to be crashed due to loss of genetic diversity, the accumulation of deleterious mutations, and changes in genetic make-up resulting from genetic drift and increased inbreeding^1,2^. Establishing gene flow between populations is an effective method to improve the fitness of isolated small populations by increasing genetic diversity and decreasing inbreeding, which is also known as the genetic rescue^3–5^. For example, gene rescue substantially improved the fitness of small inbred populations in African lions (*Panthera leo*)^6^, mountain pygmy possum (*Burramys parvus*)^4^ and Florida panther (*Puma concolor coryi*)^7^. However, the making of suitable strategy for genetic rescue highly depends on comprehensive investigation of the genetic background of these small populations, including the genetic diversity, population differentiation, mutational load, gene flow, local adaptation, the extent of inbreeding and outbreeding, etc^2,8,9^.

Molecular markers have ever been widely used in conservation genetics to guide the protection and conservation of threatened species, with the most promising markers of microsatellite and mitochondrial DNA^10–12^. However, limitations of these molecular markers are also obvious, including the limited information to explain local adaptation, inbreeding/outbreeding depression and adaptive variations^13^. Bias introduced into parameter estimations due to the limited genetic information is also needed to be emphasized for the used of neutral markers^14,15^, and the evaluation of genetic diversity of the giant panda (*Ailuropoda melanoleuca*) is a good example for this issue^16^. Fortunately, all the above-mentioned limitations could be much improved at the era of whole genome sequencing. With the rapid development of sequencing technology and the plummeting cost of re-sequencing, the conservation genetics is in transition to conservation genomics^17^. Recently, several genomic-based studies on investigation of endangered animals were reported, explaining many long-standing questions, such as the purging of deleterious mutations in Bengal tigers (*Panthera tigris tigris*)^18^, the local adaptation of giant panda^19^, the detailed inbreeding depression in kākāpō (*Strigops habroptilus*)^20^, which are unthinkable without application of genome-wide markers.

Pangolins belong to the placental mammal order of Pholidota, representing one of the most unusual orders of mammals due to their overlapping epidermal scales, myrmecophagous diet, lack of teeth, as well as their extraordinarily elongated tongue^21,22^. They play important roles in ecosystems, including predators of social insects, creators of burrows, hosts of endo- and ectoparasites, and also prey for other predators^23–25^. Humans, on the other hand, have extensively killed and exploited pangolins as a luxury delicacy and used their scales in traditional medicines^26–28^. Because of this overexploitation, all the eight extant Pholidota species have undergone severe population decline^29^. Of all eight pangolin species, the Chinese pangolin (*Manis pentadactyla*) is one of the most threatened species with a ~94% decline in its whole population between the 1960s and 1990s, largely due to the extensively illegal trade in Asia^30,31^. It has been classified as a critically endangered species in the IUCN Red List of Threatened Species and listed in ‘Appendix I’ of the Convention on International Trade in Endangered Species of Wild Fauna and Flora (CITES)^32^. This species was once distributed in the vast areas of the southern Yangtze River in China and some northern areas of Southeast Asia^30,33,34^. However, new burrows have not been seen for more than 20 years in some areas of the Dawuling Natural Reserve and Luofushan Natural Reserve in Guangdong province^35,36^. Moreover, the Chinese pangolin might have been extirpated from several areas, including Jiangsu, Shanghai, Henan, etc^37,38^.

A large majority of research on pangolins is restricted to ecological study or genetic study but with DNA markers limited at mitochondrial DNA and microsatellite fragment^38–40^. Recently, Hu et al.^41^ reported the population identities of illegally traded individuals, revealed population fluctuations, and an increase in inbreeding and mutation load in Chinese pangolin populations, providing valuable information for the conservation of this species. Further, great progress has been made towards the conservation of Chinese pangolin, with several Chinese pangolins being photographed by infrared cameras in the wild in at least six cities of Guangdong province since 2020^42^, which was a promising sign for the rescue and recovery of this species. Although, Hu et al.’s study included samples from Taiwan^43^, Yunnan province, and some unknown geographical locations, more samples from Guangdong province are needed for conjoint analysis to reveal more comprehensive genomic make-ups of this critically endangered species.

The planning of genetic rescue necessitates a thorough assessment of genetic background from a variety of perspectives, which is the prime objective of our research to aid in the continued conservation of Chinese pangolins. In this study, we intensively investigated the genomic characteristics of Chinese pangolins from Guangdong province. Together with previously published genomes, we revealed the population structure of the Chinese pangolins, and extensively explored the genetic diversity, population differentiation, mutational load, gene flow and local adaptations of each population. Our study provides a valuable resource and insights for the future conservation and conservation of this endangered species.

## Results

### Characteristics of sequencing data and variants

The sequencing depth of 15 Chinese pangolin individuals ranged from 13.17 X to 22.85 X, with an average depth of 17.03 ± 2.43 X (Supplementary Table 1). Together with the published genome sequencing data from other 22 individuals, we obtained 40,855,034 raw single-nucleotide polymorphisms (SNPs) for the whole pangolin population. After deep filtration, 35,023,399 SNPs were retained for further analysis. As expected, most of the SNPs were distributed in the intergenic regions. The distribution of SNPs against the minor allele frequency (MAF) showed that the proportion of SNPs with a minor allele frequency less than 5% (20,155,038 SNPs) was 49.3%, representing a large proportion of low-frequency variants in the pangolin population (Supplementary Fig. 1). In addition, we also observed significant variability in the SNP number of different populations (*P* = 7.5 × 10^−11^ ~ 1.1 × 10^−12^) (Supplementary Fig. 2).

### Genetic relationships among Chinese pangolin populations

**Fig. 1:**
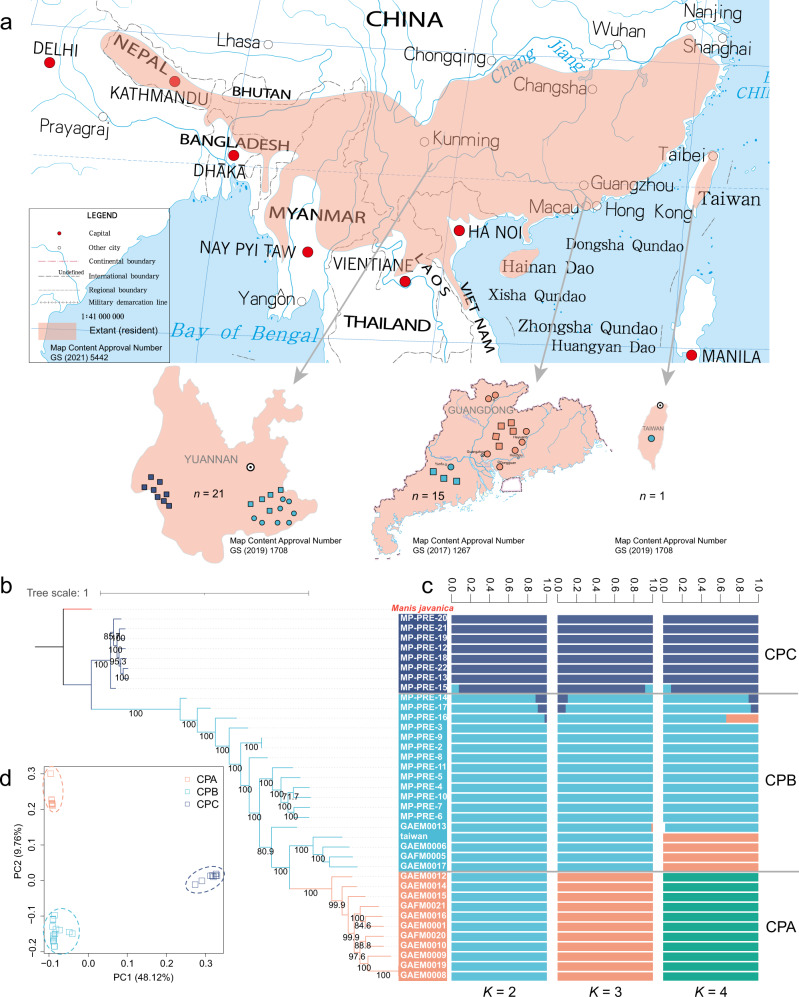
Distribution and population structure of Chinese pangolins in southern China. **a** Distribution of extant Chinese pangolins and 37 individuals in the current study. The distribution range of extant Chinese pangolins was from IUCN^29^. Pink regions represent areas where Chinese pangolins have been recorded. Circles represent samples with known origins. Squares represent the samples of unknown geographical information. CPA (*n* = 11), CPB (*n* = 18) and CPC (*n* = 8) was in orange, blue and purple, respectively. **b** Maximum likelihood phylogenetic tree of Chinese pangolin populations. Numbers at tree nodes represented the bootstrap supports (1000 replications). The Malayan pangolin, CPA, CPB and CPC are in red, orange, blue and purple, respectively. **c** Population structure inferred using ADMIXTURE analysis from K = 2 to K = 4. Clusters were noted by different colors, and each bar represented an individual. **d** Principal component analysis of all 37 individuals.

We first constructed phylogenetic trees with nuclear genomes and mitochondrial gene sequences and confirmed that these Guangdong individuals are Chinese pangolins (Supplementary Figs. 3–4). We then extensively explored the genetic relationships of 37 Chinese pangolins in the whole-genome level. In general, results from principal component analysis (PCA), admixture and phylogenetic tree supported that CPA, CPB and CPC populations were assigned to three distinct clusters (Fig. 1b–d). Fifteen individuals from Guangdong province were distributed into two clusters, with one formed by only Guangdong individuals (CPA: *n* = 11) and the other one (CPB: *n* = 18) consisting of individuals from Guangdong (*n* = 4), Taiwan (*n* = 1) and Yunnan (*n* = 13). CPC population (*n* = 8) was made up of 8 individuals who might have originated from Myanmar^41^.

### Genetic differentiation and Gene flow among populations

**Fig. 2:**
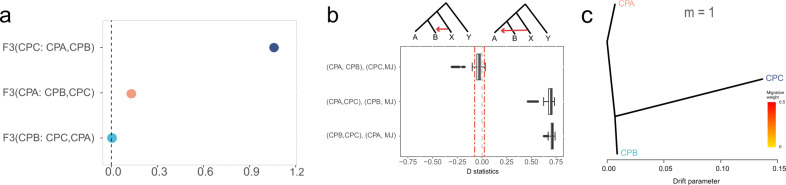
Genetic differentiation and gene flow analysis among Chinese pangolins. **a** F3 statistics for CPA, CPB and CPC. **b** D-statistics: A, B, X were among each of three Chinese pangolin populations and the Malayan pangolin (MJ) was set as the outgroup (Y). The red dashed lines represent the threshold value of D that is significantly deviating from 0. **c** TreeMix trees with no migration events (m = 1).

Genome-wide population differentiation further revealed that the CPA was distinct from the CPC with an extremely high fixation index (*F*_ST_) value of 0.541. The *F*_ST_ between the closer CPA and CPB populations was still larger than 0.1 (Supplementary Fig. 5). The F3 statistics also supported that none of these three pangolin populations was admixed by the other two populations with positive scores in each combination (Fig. 2a).

To explore possible genetic exchanges among these Chinese pangolin populations, we performed ABBA-BABA test to quantify the shared derived alleles between populations. We set the Malayan pangolin (*Manis javanica*) as the Y (the outgroup). A, B and X were from all possible combinations of the CPA, CPB and CPC populations. Interestingly, when the X population was set to be the CPC population, we were unable to detect any significantly unbalanced derived allele sharing, supporting the large differentiation between CPC and other two populations (Fig. 2b). We also performed a TreeMix analysis to further quantify gene flow among populations. However, no migration events found among the three populations with tests from m = 1 to m = 10 (Fig. 2c).

Further, identity-by-decent (IBD) analysis showed no large segments (>1 Mb) among populations. For medium-size (100 kb < IBD < 1 Mb) segments, we identified 0.81 Mb shared between CPA and CPB, 0 Mb shared between CPA and CPC, and 4.44 Mb shared between CPB and CPC (Supplementary Fig. 6). However, much more and larger IBD segments were found within populations (Supplementary Fig. 7). The total length of IBD in CPA, CPB and CPC were 251.75 Mb (10.49%), 51.45 Mb (2.14%) and 48.38 Mb (2.02%), respectively.

### Population separation among CPA, CPB and CPC populations

**Fig. 3:**
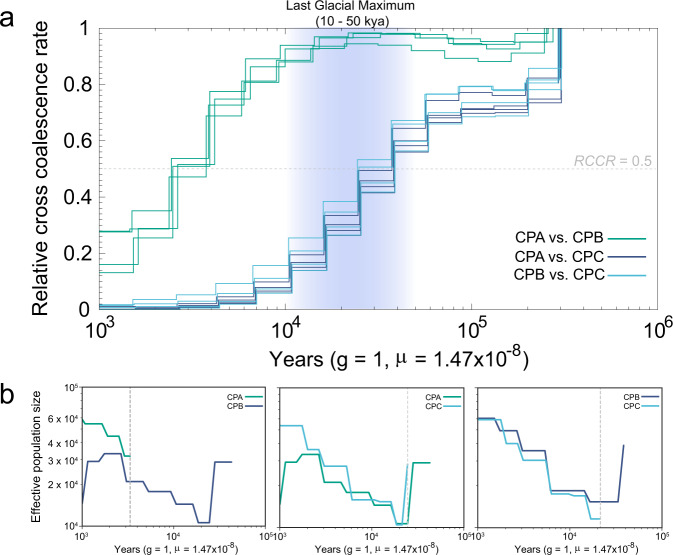
Population separation inferences in each pair of Chinese pangolin populations. **a** The split time of CPA vs. CPB, CPA vs. CPC, and CPB vs. CPC populations was calculated by the MSMC2 software. Each line indicated a pair of separation inferences by randomly selecting two individuals. The grey dashed line represented that relative cross coalescence rate (RCCR) of 0.5. The Last Glacial Maximum period is indicated by purple vertical bars. **b** The split time between CPA vs. CPB, CPA vs. CPC, and CPB vs. CPC populations inferred by the SMC++ software. The lines represent the CPA (green), CPB (purple) and CPC (blue) populations. The grey dashed line denotes the divergence between two populations.

Considering multiple lines of evidence from population structure and gene flow, we inferred that the separations between CPA and CPB were more recent than that of CPC and CPA/CPB. As we expected, the relative cross coalescence rate (RCCR) estimation showed that the separation between CPA and CPB was 2.5 to 4.0 thousand years ago (kya), which was much more recent than that between CPC and CPA (25–40 kya), and CPC and CPB (25–40 kya) (Fig. 3a). Considering the possible phasing errors which could influence the accuracy of the MSMC2 method, we further performed SMC++ to validate results from MSMC2. Again, we found that the CPA and CPB populations were separated at ~ 3.4 kya, CPC was separated from CPA and CPB at ~ 25 kya (Fig. 3b), which supported results from MSMC2.

### Genetic diversity and inbreeding

The richness of genetic diversity in a population reflects its evolutionary potentials to adapt to environmental changes^44^. The average heterozygosity (*He*) among all the individuals was 0.18% calculated by all SNPs. This value decreased to 0.15% when low-frequency alleles were excluded (allele frequency (AF) < 5%). To better compare our results to the previous report, we also calculated the average *He* (0.11%) using SNPs with AF > 20% (Supplementary Fig. 8a). Similar patterns were also found in nucleotide diversity (π), with the CPB population having the highest level of genetic diversity (Supplementary Fig. 8b).

**Fig. 4:**
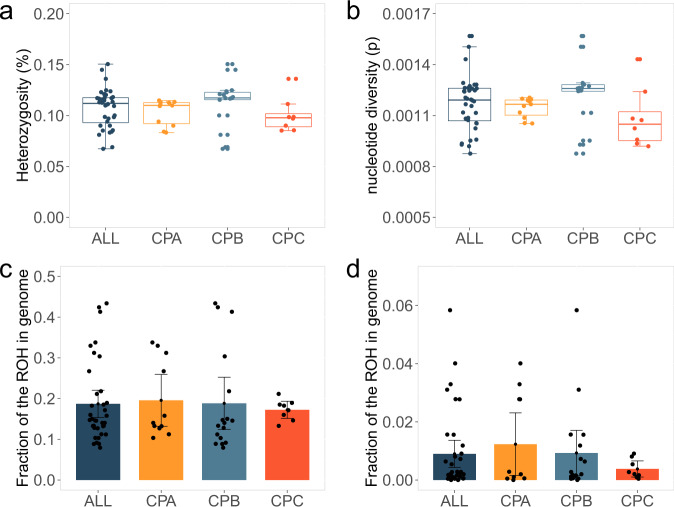
Genetic diversity and ROH distribution in the Chinese pangolin genome. **a** Heterozygosity in each population. **b** Nucleotide diversity in each population. Error bars show range of values within 1.5 times the interquartile range. **c**, **d** Fraction of run of homozygosity (ROH) in the genome of each population, including the medium (**c**: 100 kb–1 Mb) and long (**d**: > 1 Mb) ROHs. Data are presented as mean ± SD. ALL: *n* = 36. CPA: *n* = 11. CPB: *n* = 17. CPC: *n* = 8.

By using the same method from Hu et al.^41^, we found 77,251 runs of homozygosity (ROHs) ranging from 100.0 kb to 3155.8 kb in the whole Chinese pangolin population. Of these ROHs, 448.58 Mb (F_ROH_ = 18.69%) and 21.57 Mb (F_ROH_ = 0.90%) were assigned to medium-size (100 kb–1 Mb) and long ROH ( > 1 Mb) (Fig. 4c, d; Supplementary Figs. 8c, d). The medium-size ROHs in CPA, CPB, and CPC population was 469.38 Mb (F_ROH_ = 19.56%), 451.63 Mb (F_ROH_ = 18.82%), and 413.52 Mb (F_ROH_ = 17.23%), respectively. The long-size ROHs in CPA, CPB, and CPC population was 29.54 Mb (F_ROH_ = 1.23%), 22.28 Mb (F_ROH_ = 0.93%), and 9.11 Mb (F_ROH_ = 0.38%), respectively.

### Mutational load

On average, we found 266 ± 55 deleterious nonsynonymous SNPs (nsSNPs) across all three populations (Supplementary Fig. 9a). Individuals in the CPC population (169 ± 15) exhibited a significantly smaller number of deleterious nsSNPs when compared to CPA (287 ± 15; *P* = 1.7 × 10^−10^) and CPB (298 ± 20; *P* = 8.5 × 10^−12^) populations. However, there was no significant difference in the ratio of deleterious nsSNPs to total nsSNPs across all populations (*P* = 0.8~0.9; Supplementary Fig. 9b). We also considered the number of Loss of Function (LoF) variants in each population (from 785 to 1250; Supplementary Fig. 9c). The number of LoF variants in CPC population was significantly lower than CPA and CPB populations (*P* = 1.4 × 10^−7^ and *P* = 5.5 × 10^−9^). However, the CPC population had a significantly higher ratio of LoF variants to total nsSNPs than CPA and CPB populations (*P* = 8.5 × 10^−8^ and *P* = 2.9 × 10^−8^; Supplementary Fig. 9d).

**Fig. 5:**
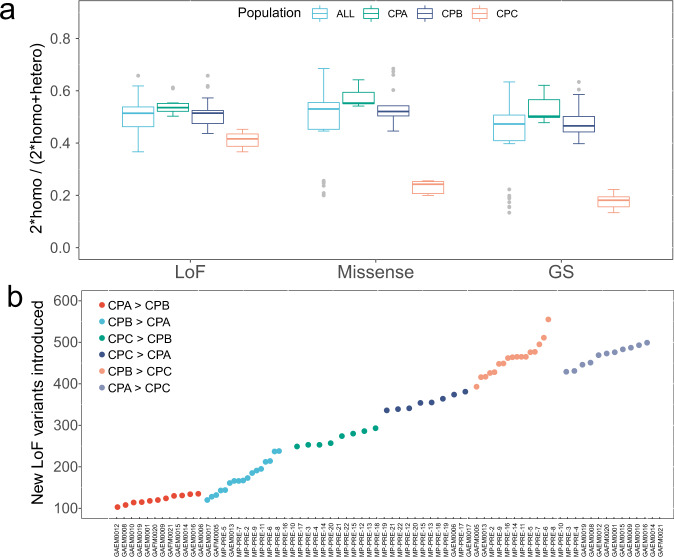
Mutation load estimation for the pangolin population. **a** The derived mutations in the coding region include Loss of Function (LoF) mutations, missense mutations, and deleterious missense mutations (GS). Error bars show range of values within 1.5 times the interquartile range. ALL: *n* = 36. CPA: *n* = 11. CPB: *n* = 17. CPC: *n* = 8. **b** The predicted number of new LoF variants introduced between individuals from different populations if translocation took place.

We further evaluated the level of mutational load by measuring the number of homozygous sites to the homozygous and heterozygous sites (hereafter ratio) for LoF mutation, missense mutation, and deleterious missense mutations. We found that this ratio of LoF mutation, missense mutation, and deleterious missense mutations in the 36 Chinese pangolins was 0.505, 0.482 and 0.432, respectively (Fig. 5a). The ratio of LoF mutation (0.545, 0.522), missense mutation (0.575, 0.540), and deleterious missense mutations (0.533, 0.485) in CPA and CPB population were very similar, but significantly higher than that in the CPC population (0.412 for LoF mutation, 0.232 for missense mutation, and 0.178 for deleterious missense mutations) (Fig. 5a, Supplementary Table 4).

We further evaluated the number of potentially introduced LoF mutations to the receiving population from the donor population. We found that CPB individuals would introduce 120–238 LoF mutations and CPC individuals would introduce 336–381 LoF mutations when CPA was regarded as the receiving population. The minimum LoF mutations (103–135) would be introduced to the CPB population from the CPA population. However, this number rise to 393 to 555 by introducing CPA and CPB individuals to the CPC population (Fig. 5b).

We then analyzed the 2229, 1044, 1574 and 962 genes affected by the LoF mutations in the all 36 individuals, CPA, CPB and CPC populations. In general, significantly enriched Gene Ontology (GO) categories and Kyoto Encyclopedia of Genes and Genomes (KEGG) pathways indicated that these genes were associated with male gamete generation (GO: 0048232; *P* = 3.8 × 10^−13^), visual perception (GO:0007601; *P* = 8.8 × 10^−10^) and protein digestion and absorption (KEGG: hsa04974; *P* = 3.0 × 10^−6^) (Supplementary Tables 5–8). The KEGG pathway “protein digestion and absorption (*P* < 0.01)” was found to be significantly enriched in all three populations. In addition, the Insulin signaling pathway (KEGG: hsa04910; *P* = 6.4 × 10^−5^), cellular carbohydrate metabolic process (GO: 0044262; *P* = 1.9 × 10^−4^) and response to fructose (GO:0009750; *P* = 2.0 × 10^−4^) were found in the CPB population. In particular, we did not find enriched gametogenesis-related pathways, such as male gamete generation, in the CPC population.

### Recent positive selection in Chinese pangolin populations

A total of 2218 (CPA), 3154 (CPB) and 1878 (CPC) SNPs were identified under strong positive selection with iHS scores in the top 0.1% (Supplementary Fig. 10). By filtering out SNPs in the non-gene regions, 176, 299 and 277 genes were located in the positively selected regions for CPA, CPB and CPC populations, respectively. Eleven genes were shared among these three populations, but 130, 226 and 219 genes were unique to CPA, CPB and CPC populations, respectively. Enrichment analysis on these selected genes showed that 14, 13 and 16 GO categories or KEGG pathways were unique for CPA, CPB and CPC populations, respectively. These categories were correlated with various biological functions including cellular processes, biological regulation and metabolic processes (Supplementary Fig. 11).

## Discussion

Here we report the, to the best of our knowledge, first genetic survey at the whole genome scale for the Chinese pangolin from Guangdong province of China. We thoroughly examined the population structure of Chinese pangolins by combining Guangdong samples and published sequences, which presumably cover the majority of the distribution area of this species. We systematically investigated genomic make-ups of long-term declined Chinese pangolins, especially for the Guangdong population, including the genetic diversity, ROH and mutational load assessment, which is expected to support the future conservation and genetic rescue of this species.

Although two pangolin individuals in Hu et al.’s study (MP09 and MP10) were mentioned to be possibly from Guangdong province, the detailed population structure is hard to be further described due to the ambiguous sampling site and small sample size^41^. Benefiting from the relatively large sample size collected from several sites in Guangdong province, we defined a new Chinese pangolin population, CPA, in our study from the prospective of genomics, with multiple lines of evidence (Fig. 1b–d). Negligible gene flow further strengthens the independent state of the CPA population (Fig. 2).

A very large genetic differentiation between this CPA and CPC (*F*_ST_ = 0.541) population suggested a potential subspecies-level divergence when compared with other animals. For example, the Sichuan and Qinling giant panda subspecies was defined in 2005 by the skull and molar size^45^, and their genomic differentiation is much less than the CPA and CPC populations (*F*_ST_ = 0.140)^16^. The differentiation of tiger subspecies could be dating back to 110 kya^46^, however, the genomic differentiations among tiger subspecies are still much lower than these two Chinese pangolin populations, with the highest *F*_ST_ value between Amur tiger and Sumatran tigers is of 0.318^47^. This was not surprising, because the CPC population was significantly distinct from CPA and CPB from the perspective of genetics (Supplementary Fig. 5), which was also found in Hu et al.’s study with the *F*_ST_ of 0.495 between MPA and MPB^41^.

Interestingly, the genomic differentiation between CPA and CPB was moderate with a *F*_ST_ value of 0.101. Although this differentiation was much lower than that between CPA and CPC, this extent was comparable with differentiations between populations with extensive geographic separations, like the divergence of African leopard (*Panthera pardus*) populations with the average *F*_ST_ value of 0.104^48^, the two largest isolated populations (Minshan and Qionglai populations) of the giant pandas^16^, and even comparable to that between African and Asian humans (*F*_ST_ = 0.120)^49^ which are known to be separated before ~ 55 kya^50^. Taken together, we infer that the first and newly discovered CPA was a distinct Chinese pangolin population, and suggested an extra conservation unit that is parallel with CPB and CPC population. We believe the future large-scale population genomic analysis and corresponding ecological and morphological studies will further enrich and strengthen this claim.

We inferred the separation between CPA and CPB was much more recent than that between CPC and CPA/CPB (Fig. 3a), which is logically consistent with population structure analysis. The separation between CPC and CPA/CPB was coincided closely with the last glacial period (LGP, ∼10–120 kya), especially the beginning of the last glacial maximum (LGM, ∼10–50 kya)^51,52^. The extremely cold weather and the possibly continuous impact on the prey of Chinese pangolins during this period could be one of the reasons for population separation, considering that southern China was also severely influenced by the LGP^53^. However, climate changes may not be the main reason for population separation^19,54,55^. Human activity is always considered an important factor for shaping the evolution of animals^9,36,56^. The human expansion in East Asia occurred as early as 40 kya^57^, and frequent migration, admixture and replacement also occurred at the original distributed area of the Chinese pangolins^41^. Interestingly, the Han Chinese population began to expand and separated from the European human population at ∼30 kya^58,59^. Moreover, the key period in human history was the onset of the Holocene since the development of more favorable climate conditions^60^. We concluded that human activity was most likely responsible for the separation of the CPA and CPB populations.

Regarding the genetic status of Chinese pangolin in this study, we observed that genome-wide *He* among the three populations are similar (0.107% ± 0.086%; *P* = 0.058~0.755), but lower than some other critically endangered species that are suffering from long-term population decline as the Chinese pangolin, such as Northern white rhinoceros (*Ceratotherium simum cottoni*) (0.110%) and Western lowland gorilla (*Gorilla gorilla gorilla*) (0.144%)^2,61^. Such a low level of genetic diversity suggested that Chinese pangolins likely had a relatively low adaptive potential^62^. The inbreeding often contributes a lot to the decrease of genetic diversity for endangered species, such as the ROH segments of highly inbred grey wolves (*Canis lupus*) population ranging from 2,695 bp to 95.8 Mb^63^ and the modern Malay Peninsula population of Sumatran rhinoceros (*Dicerorhinus sumatrensis*) estimating 30% of the genomes contain longer ROH segments (≥ 2 Mb)^8^. However, ROHs in the Chinese pangolins are found to be much shorter than the above-mentioned endangered species. We infer that the low genetic diversity in the Chinese pangolin may not from the extensively recent inbreeding due to the absence of large size ROHs^64^, but the long-term isolation without frequent gene flow between subpopulations could be an alternate explanation. Here we cannot exclude the bias introduced from the reference genome, because the accurate evaluation of ROH highly depends on the contiguity of the reference genome^65^, but no long-read assembled high-quality reference genomes are available now.

The decrease of fitness in a small population can accelerate by the accumulation of deleterious mutations^66,67^. When focusing on Guangdong populations, the ratio of deleterious mutations in CPA and CPB populations was higher than CPC population. LoF mutations enriched in pathways related to male gamete generation in the CPA and CPB populations, but not CPC population, further indicated more genetic burdens in Guangdong populations, considering the impaired gamete quality may reduce reproductive success and further affect species fitness and survival^68^. Together with the overall distribution of deleterious mutations, we speculated that the CPC population has higher fitness than both CPA and CPB populations.

LoF mutations were also found significantly enriched in the protein digestion and absorption pathway (*P* < 0.01) in all the three populations, revealing a potentially weak ability of digestion and absorption of protein in the whole Chinese pangolin population. In the CPB population, we also found enriched KEGG pathways related to carbohydrates metabolism, indicating an inferior performance in the energy supply. Considering that pangolins enjoy high protein, high fat, high calorie food^69,70^, the possibly weakened ability to use the protein and carbohydrates may further lower the fitness of the Chinese pangolins.

If a certain species has several isolated populations under different environments, local adaptation in different directions could occur in different populations^71^. Genetic rescue by populations with large differentiation shaped by natural selection tends to be disturbed by outbreeding depression^72^. To better identify the local adaption in each population, we performed enrichment analysis of genes under recent natural selection in the three populations. A large proportion of enriched pathways were unique for the three populations (Supplementary Fig. 11), indicating an obvious adaptive difference in CPA, CPB and CPC populations. It was worth noting that much of the Yunnan province lies within the subtropical highland, while Guangdong province faces the South China Sea to the south with a humid subtropical climate^73^. We inferred that the local adaptation in a different direction might have occurred in these three populations. However, more research works are needed to explore how these genes under natural selection affect the survival of this species.

In addition, we predicted an average of 309 LoF mutations could be introduced into other populations as new deleterious alleles when conducted translocations among CPA, CPB and CPC populations (Fig. 5b). The number was several dozen times higher than that predicted among three Sumatran rhinoceros populations (an average of 10 new LoF variants)^8^. We also noticed that more LoF variants could be mutually introduced between CPA/CPB and the CPC population, which is consistent with the result from local adaptation, possibly due to the long-term isolation and adaptation to their local habitats (Yunnan and Guangdong). We cannot conclude that it is 100% not suitable to implement genetic rescue among these Chinese pangolin populations, however, genetic rescue between CPA and CPB should be safer from the perspective of outbreeding depression. The conclusion will be more convincing only if the exact effects of these LoF mutations on genes could be parsed in future works.

We should keep in mind that genetic rescue is a complicated process^4,5,74^, hence more reliable evidence is still needed to be put forward in further studies. Furthermore, as a distinct population separated from the CPB and CPC, the newly discovered CPA Chinese pangolin population in our study should be given high priority in future conservation works of the Chinese pangolin.

## Methods

### Samples and data collection, library preparation, and sequencing

Approvals of all necessary research ethics and permits were granted by the Institutional Review Board of BGI (BGI-IRB E21056). 15 Chinese pangolin samples were collected from the Guangzhou Wildlife Rescue Center, Guangdong province, China for whole genome sequencing. All samples were taken from rescue individuals dead of natural causes or confiscated individuals from forestry police, and all individuals were identified as Chinese pangolins through distinct morphological characteristics. In addition, sequencing data of 22 Chinese pangolin individuals (BioProject: PRJNA529540 and PRJNA20331; Supplementary Table 2) and 72 Malayan pangolin individuals were downloaded from NCBI (BioProject: PRJNA529540; Supplementary Table 3) for downstream analysis in this study.

Genomic DNA was isolated using standard phenol/chloroform-isoamylalcohol extraction^75^ and the precipitate was solved in 20–100 μl distilled water. ~1 μg genomic DNA was used and sheared into fragments with 200 to 800 base pairs (bp) for paired-end (PE) DNA library construction with the insert size of ~350 bp following the manufacturer’s instructions of BGISEQ platform (BGI, Shenzhen, China). DNA libraries were then subjected to the DNBSEQ-T1 sequencer for sequencing.

### Genome mapping, variants calling and filtering

Sequencing reads were mapped to *M. pentadactyla* reference genome (YNU_ManPten_2.0, Genbank: GCA_014570555.1) and *M. javanica* (YNU_ManJav_2.0, Genbank: GCA_014570535.1)^41^ by using the Burrows-Wheeler Aligner (BWA) with the *mem* algorithm^76^ (version: 0.7.10–r789) using the default parameters. Sorting, reordering and reads deduplication were performed by the Picard tools (http://picard.sourceforge.net) (version: 2.1.1). HaplotypeCaller implanted in the Genome Analysis Toolkit (GATK, version: 3.3-0-g37228af)^77^ was used for raw variants calling. Hard filtering was performed on the raw SNP variant set with parameters of “QUAL < 30.0 ||  QD < 2.0 ||  FS > 60.0 ||  MQ < 40.0 ||  MQRankSum < −12.5 ||  ReadPosRankSum < −8.0”. The final high-quality SNP set was used for further genetic analyses. Lastly, SNPs in scaffolds with length smaller than 100 kb were excluded.

SNPs were annotated by the software ANNOVAR^78^ (version: 2015-12-14) with the genome of *M. pentadactyla* reference genome (YNU_ManPten_2.0). We sorted out the basic information of the resequencing data of each population (CPA, CPB and CPC) and grouped SNPs in different categories by results from ANNOVAR, including exotic, nonsynonymous, synonymous, UTR, intronic, intergenic, splicing, and non-coding RNA (ncRNA). In addition, we draw the distribution of MAF for all individuals. We prepared three SNP sets by controlling the filtration on the allele frequency for downstream analysis, including: 1) SNPs without any filtration of allele frequency; 2) SNPs with the allele frequency larger than 5%; 3) SNPs with the allele frequency larger than 20% following the method of Hu et al.^41^.

### Phylogenetic tree, PCA and admixture analysis

To avoid closely-linked sites, PLINK^79^ (version: 1.90b3.38) was used to produce a pruned subset of SNPs by Linkage disequilibrium (LD) values, resulting in a set of 171,405 SNPs. This SNP set was then converted into PHYLIP-format file by using an in-house Python script to construct the Maximum-Likelihood (ML) phylogenetic tree with 1000 replications by the PhyML^80^ (version: 20151018) with all 37 samples. The Malayan pangolin (YNU_ManJav_2.0) was used as an outgroup.

For species identification, the Chinese pangolin genome (YNU_ManPten_2.0) and Malayan pangolin genome (YNU_ManJav_2.0) was regarded as the reference genome for 109 pangolins (37 Chinese pangolin individuals and 72 Malayan pangolin individuals), respectively. We then constructed two ML phylogenetic trees according to the above methods. We also extracted the cytochrome *b* (Cyt *b*) and cytochrome *c* oxidase subunit 1 (CO1) sequences from *Felis catus*: NC_001700.1 and 8 species of *Manis* (*Manis tricuspis*: NC_026780.1, *Manis tetradactyla*: MG196299.1, *Manis gigantea*: MG196303.1, *Manis temminckii*: KP306516.1, *M. pentadactyla*: MG196307.1, *Manis crassicaudata*: NC_036433.1, *Manis culionensis*: NC_036434.1, and *M. javanica*: KT445979.1), 37 Chinese pangolin individuals and 72 Malayan pangolin individuals. ML trees with 1000 replications based on mitochondrial gene sequences were constructed using Molecular Evolutionary Genetics Analysis (MEGA, version: 10) with the Kimura’s two-parameter model^81^.

All SNPs without pruning and AF filtering were used as the initial input data for PCA and ADMIXTURE analysis. The Genome-wide Complex Trait Analysis^82^ (GCTA, version: 1.92.2) software was used for PCA inference. The population genetic structure was then inferred by using the same dataset as PCA analysis with the ADMIXTURE^83^ (version: 1.3) program. We predefined the number of genetic clusters (K) from 1 to 10 and ran the cross-validation error (CV) procedure to explore the best K, using the default parameters and settings.

### Population differentiation and gene flow

We used Weir and Cockerham’s *F*_ST_^84^ to estimate the population differentiation. All bi-allelic SNPs were used for the calculation of genome-wide *F*_ST_ between each pair of the populations using the VCFtools (version: 0.1.13) software^85^.

In order to explore whether one population was admixed by the other two populations, we performed the F3 test with combinations of CPA, CPB and CPC using the ‘qp3Pop’ in the ADMIXTOOLS^86^ (version: 5.1). The EIGENSTRAT format input data was generated by CONVERTF program in the ADMIXTOOLS.

To examine the excess of shared derived alleles between different populations of Chinese pangolins, we applied the classic ABBA-BABA test (D statistics)^87^ using the “-informative” command of the POPSTATS^88^. The four populations were set to be ((A, B), (X, Y)), and the A, B and X were among each of CPA, CPB, and CPC. The Y was Malayan pangolin which was the outgroup. We screened significant D values using the Z-score (|Z | > 3) based on a block jackknife procedure.

The sharing of identity by descent (IBD) between individuals was calculated by the RefinedIBD^89^ (version: 16May19. ad5) with analysis parameters (length = 0.1 and lod = 3.0). We compared different lengths of IBD (IBD > 1 Mb or 100 kb < IBD < 1 Mb) and their percentage on the genome among populations to evaluate the degree of gene flow.

We constructed maximum-likelihood population trees using TreeMix^90^ (version: 1.13) to investigate the phylogenetic relationship in the presence of admixture events among populations. TreeMix was run with the parameters -bootstrap 5000 -global and the migration event -m (from 0 to10).

### Population separation among pangolin populations

We first used MSMC2^59^ (version: 2.1.2) to infer the separation among the three populations. MSMC2 was performed for four independent replications with two samples randomly selected from each population. Genotype phasing was using the Beagle^91^ (version: 5.0) software with default parameters before the MSMC2 inference. Parameters for MSMC2 calculations were as follow: -skipAmbiguous -I 0-4, 0-5, 0-6, 0-7, 1-4, 1-5, 1-6, 1-7, 2-4, 2-5, 2-6, 2-7, 3-4, 3-5, 3-6, 3-7 -i 20 -t 6 -p ‘10*1 + 15*2’. To further validate the results inferred by the MSMC2, we performed the SMC++ ^92^ (version: 1.5.2) to infer the split time of the two populations, because the SMC++ software does not depend on phasing data, which can avoid calculation bias introduced by switch errors during phasing analysis. The mutation rate and generation interval of the *M. pentadactyla* we used here was 1.47×10^−8^ ^41,43^ per site per generation and one year^41,93^.

### Genetic diversity and ROH analysis

VCFtools was used to estimate whole-genome genetic diversity, including heterozygosity (*He*) and nucleotide diversity (π)^94^. ROH was identified by PLINK following the method described by Dobrynin et al.^95^. Long ROH is often the indicator of recent inbreeding that occurred several decades ago. According to the formula reported by Kardos et al. (Generations = 100/2 * ROH _length_)^41,63^, we only counted ROHs that were larger than 100 kb in this study. If ROHs were longer than 1 Mb, we assumed that these ROHs were generated by more recent inbreeding (< 50 years). The genetic diversity, ROH and mutation load of Taiwan individuals have been studied in the study by Hu et al.^41^. Therefore, Taiwan individuals were excluded from this part and mutational load analysis.

### Mutational load

We used the genotypes of the same alleles in the *M. javanica* to represent the ancestral state before identifying derived mutational loads. A deleterious mutation we used here means that an amino acid change in a protein was predicted to be harmful to the function, which becomes the main genetic basis of inbreeding depression^96^. The deleteriousness of derived mutations was diagnosed using the Grantham Score (GS)^97^. Here, nsSNPs with GS ≥ 150 were defined as deleterious mutations^97,98^. We used “-aamatrixfile grantham matrix” parameter in the package ANNOVAR to print out GS for nonsynonymous variants. We counted the number of deleterious nsSNPs and the ratio of deleterious nsSNPs to total nsSNPs. Moreover, we selected derived mutations in coding regions of each pangolin individual for annotation by SnpEff^99^ (version: 4.3). LoF variants here we used included *splice_donor_variant, splice_acceptor_variant* and *stop_gained*. Numbers of LoF variants and ratios of LoF variants to total nsSNPs in Chinese pangolin populations were counted. Missense mutations were represented by *missense_variant*. The ratio of homozygous (two per site) to (homozygous (two per site) plus heterozygous sites (one per site)) for all LoF, missense and deleterious variants were calculated for estimating the level of mutational load^44^.

GO and KEGG functional enrichment of genes affected by LoF mutations was performed using Metascape website (Last modified January 1, 2022)^100^. The GO terms and KEGG pathways with an enrichment factor > 2 and a multi-test adjusted *P*-value < 0.05 were considered to be significantly enriched. *P*-values and multi-test adjusted *P*-values were transformed with log base 10.

### Recently natural selection in populations

We computed the iHS^101^ (version: 1.3) to identify genomic signatures of positive selection in the CPA, CPB and CPC populations. The iHS calculations were performed independently in each population. As the genetic distance between adjacent SNPs was needed for the calculation, a chromosome segment of 1 Mb was straightly converted as 1 centiMorgan (cM).

For the identification of candidate genes, SNPs within the top 0.1% iHS scores were assigned as candidate sites. Based on candidate sites, we then used three methods to screen candidate regions described as Voight et al.^101^: a) regions of consecutive 50 SNPs; b) regions of 100 kb with 50 kb step size; c) 5 kb flanking regions away from candidate sites. We then calculated the sum of the iHS scores (siHS score) of all candidate sites in each genome region, and candidate regions were selected with the top 10% siHS score. The intersection of candidate genes obtained by three region-selected methods were used for the next analysis^101^. For the candidate genes, GO and KEGG functional enrichment was performed using Metascape website^100^. The GO terms and KEGG pathways with an enrichment factor > 2 and a multi-test adjusted *P*-value < 0.05 were regarded as significantly enriched.

### Statistics and reproducibility

To test the significant difference of ROH and mutation load between different populations, two-sided Welch two-sample *t*-tests were performed in R^102^ (version: 4.0.2). *P*-value less than 0.05 was considered to be significant. All statistics was done using available packages and reproducibility can be accomplished using parameters we mentioned in Methods.

### Reporting summary

Further information on research design is available in the Nature Research Reporting Summary linked to this article.

## Supplementary information

Supplementary Information

Description of Additional Supplementary Files

Supplementary Data 1

Reporting Summary

## Data Availability

All data generated or analyzed during this study are included in this published article and its supplementary files. Source data underlying Figures (1bcd, 2, 3ab, 4 and 5ab) in this article were provided in Supplementary Data 1. The data that support the findings of this study have been deposited into CNGB Sequence Archive (CNSA)^103^ of China National GeneBank DataBase (CNGBdb)^104^ with accession number CNP0001723 (https://db.cngb.org/).
