# Supplementary material for: Whole-genome resequencing of Chinese pangolins reveals a population structure and provides insights into their conservation
Source: Commun Biol. 2022 Aug 25;5:821. doi: 10.1038/s42003-022-03757-3 (PMC9411537; doi:10.1038/s42003-022-03757-3)
Supplement: Supplementary file 1 — Supplementary Information [file 42003_2022_3757_MOESM1_ESM.pdf]

Supplementary Information

Whole-genome resequencing of Chinese pangolins reveals a population structure and provides insights into their conservation

Qing Wang[1, 2*], Tianming Lan[2, 3, 4*#], Haimeng Li[1, 2*], Sunil Kumar Sahu[2], Minhui Shi[1, 2], Yixin Zhu[1, 2], Lei Han[5], Shangchen Yang[6], Qian Li[1, 2], Le Zhang[5], Zhangwen Deng[7], Huan Liu[2, 3, 4#], Yan Hua[8#]

[1] College of Life Sciences, University of Chinese Academy of Sciences, Beijing, China.

[2] State Key Laboratory of Agricultural Genomics, BGI-Shenzhen, Shenzhen, China.

[3] BGI Life Science Joint Research Center, Northeast Forestry University, Harbin, China.

[4] Guangdong Provincial Key Laboratory of Genome Read and Write, BGI-Shenzhen, Shenzhen, China.

[5] College of Wildlife and Protected Area, Northeast Forestry University, Harbin, China.

[6] College of Life Sciences, Zhejiang University, Hangzhou, China.

[7] Guangxi Forest Inventory and Planning Institute, Nanning, China

[8] Guangdong Provincial Key Laboratory of Silviculture, Protection and Utilization, Guangdong Academy of Forestry, Guangzhou, China.

* These authors contributed equally to this work.

[#] Correspondence:

lantianming@genomics.cn; liuhuan@genomics.cn; wildlife530@hotmail.com.

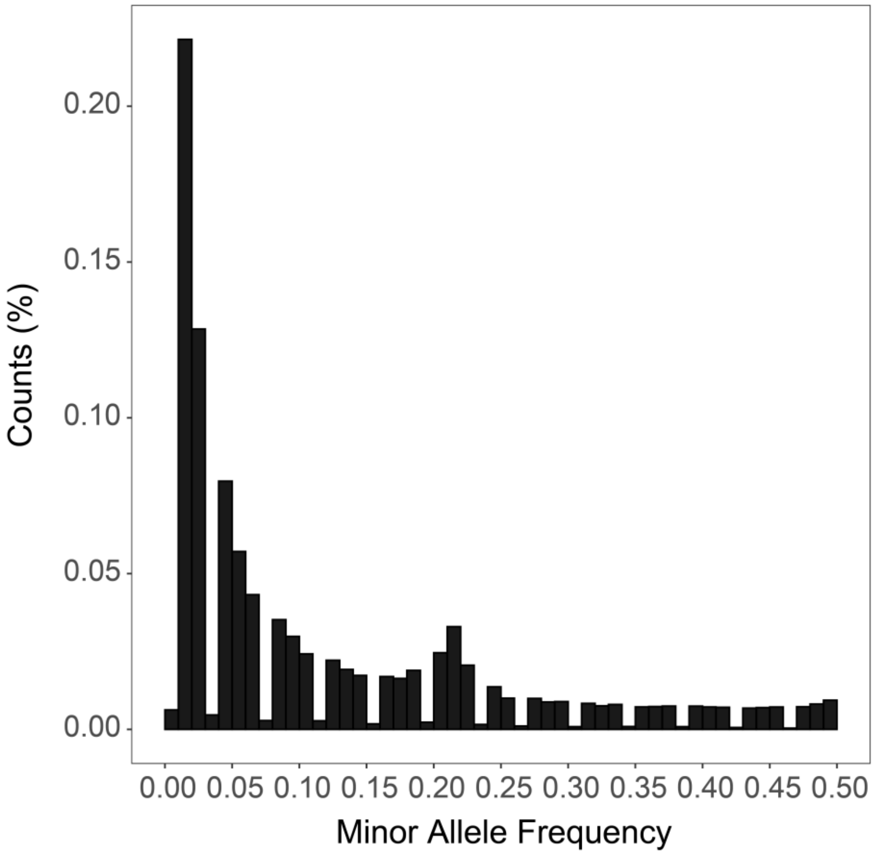

Supplementary Figure 1. The distribution of SNPs of the whole pangolin population against the minor allele frequency.

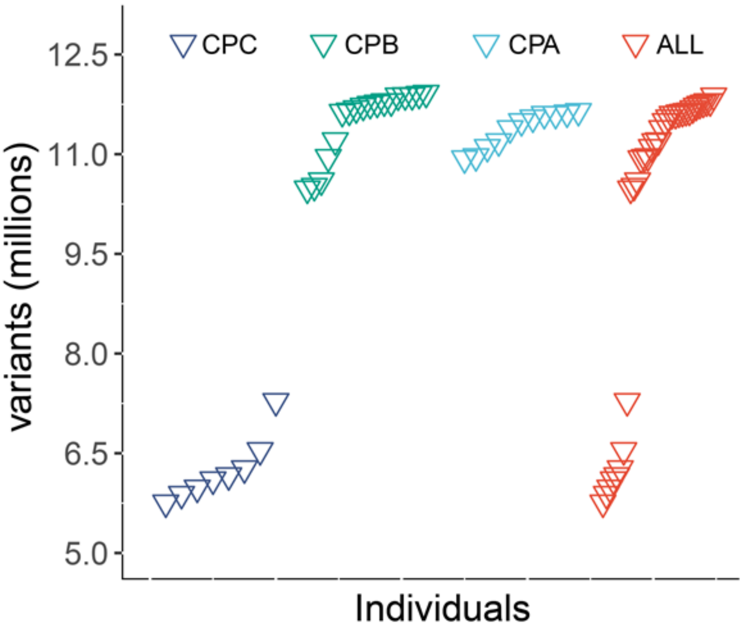

Supplementary Figure 2. SNP numbers of different populations and individuals.
Each inverted triangle represents an individual.

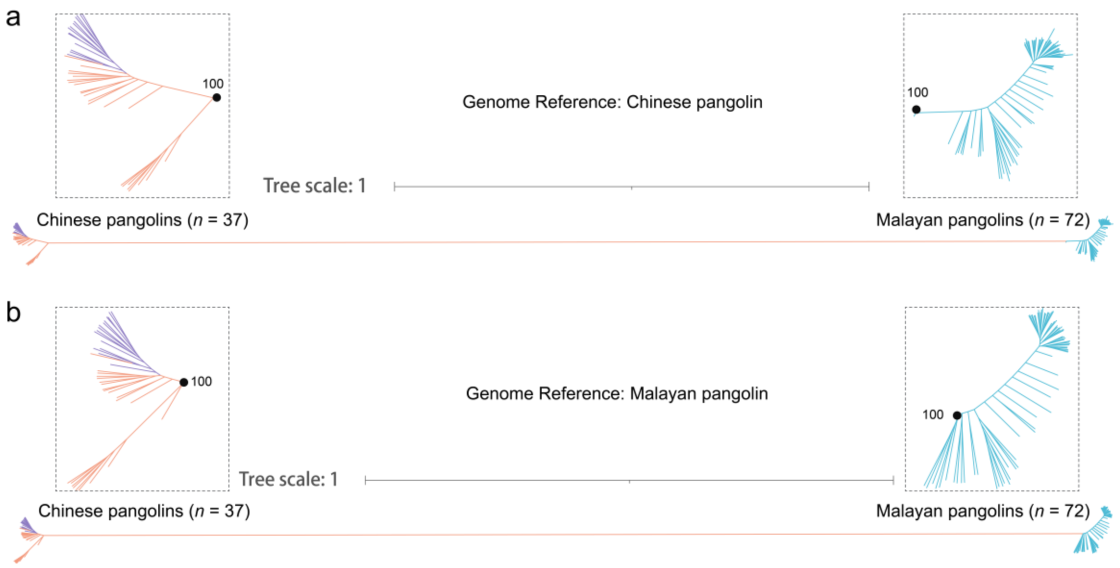

Supplementary Figure 3. Species identification by constructing unrooted Maximum Likelihood (ML) phylogenetic trees based on nuclear genome sequences. 15 Chinese pangolin individuals sequenced in the present study, 22 Chinese pangolin individuals and 72 Malayan pangolin individuals from previous studies are shown in purple, orange and blue colors, respectively. **(a)** ML tree was constructed using genome-wide SNPs based on the Chinese pangolin genome (YNU_ManPten_2.0) [1]. **(b)** ML tree was constructed using genome-wide SNPs based on the Malayan pangolin genome (YNU_ManJav_2.0) [1]. Values above the black circle represent bootstrap supports.

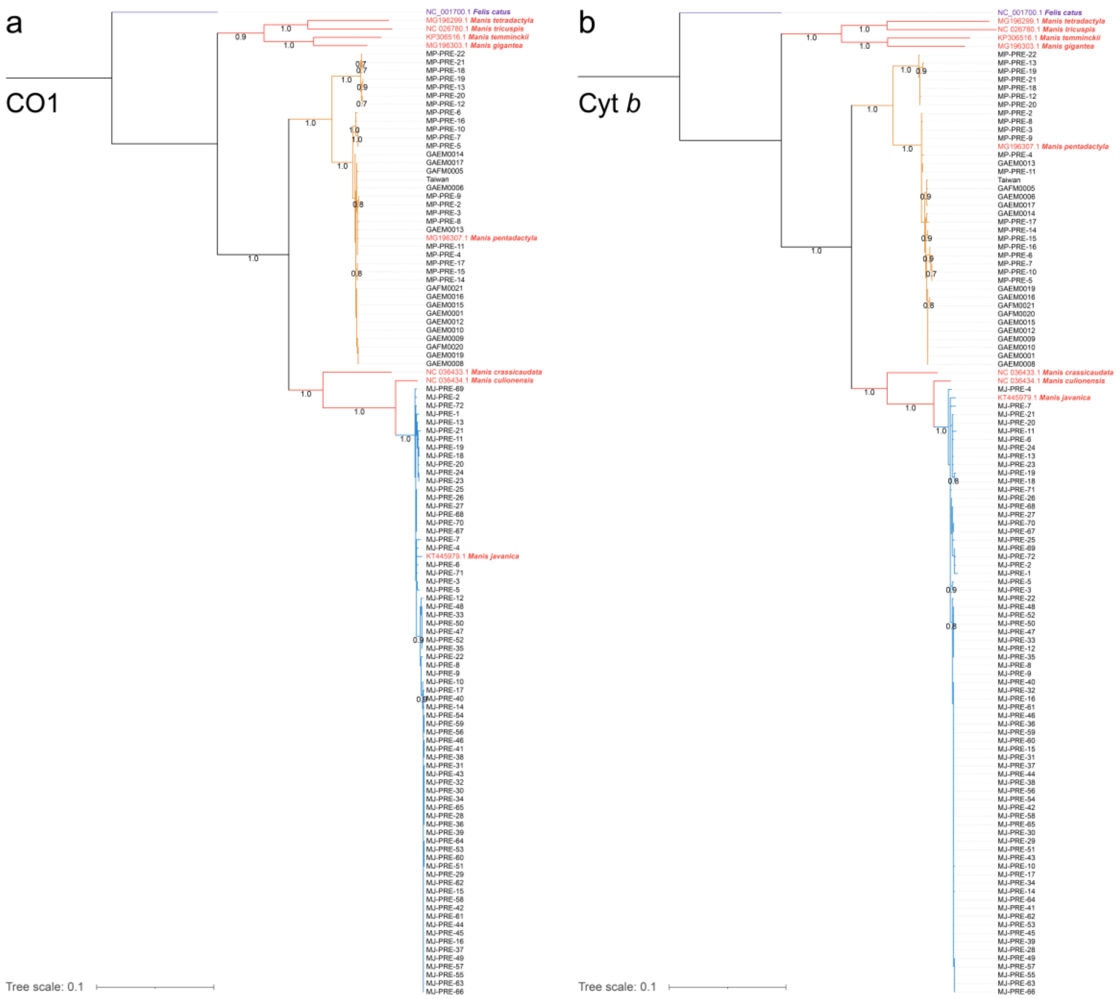

Supplementary Figure 4. Species identification by constructing of Maximum-Likelihood (ML) phylogenetic trees based on mitochondrial gene sequences. (a) ML phylogenetic tree constructed by CO1 gene. **(b)** ML phylogenetic tree constructed by Cyt *b* gene. The outgroup species, *Felis catus* is shown in purple. CO1 and Cyt *b* gene sequences from eight *Manis* species are shown in red. The orange nodes represent Chinese pangolin individuals, including 15 individuals sequenced (GA*) in this study, and 22 individuals (MP-PRE-* and Taiwan) collected from the previous study[1]. The blue nodes represent 72 Malayan pangolin individuals (MJ-PRE-*) from the previous study[1]. The bootstrap values greater than 0.7 are displayed.

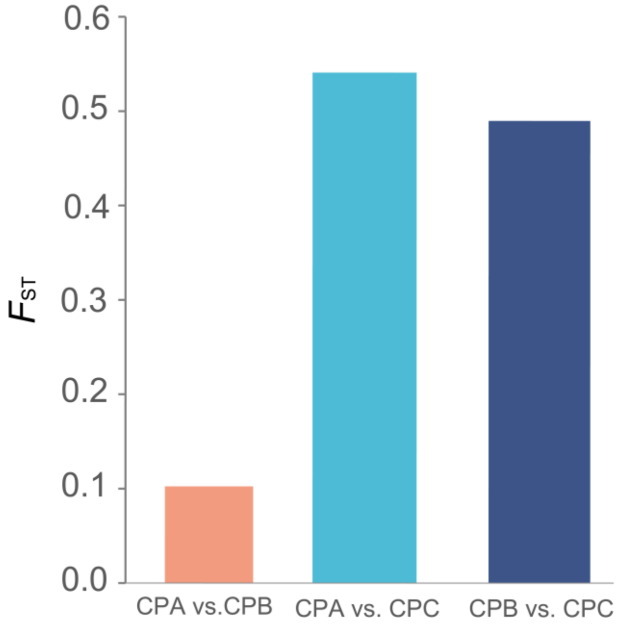

Supplementary Figure 5. Fixation index (F_{ST}) values between each pair of three Chinese pangolin populations.

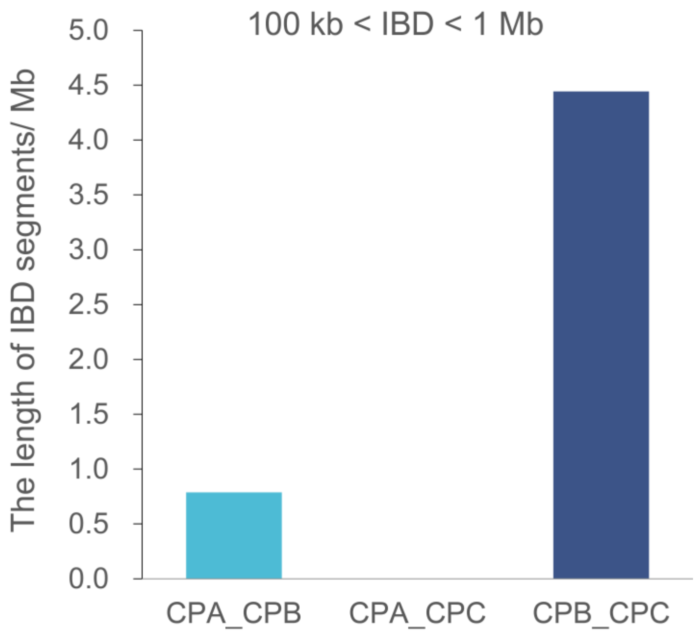

Supplementary Figure 6. The length of medium-size identity-by-decent (IBD) segments (100 kb < IBD < 1 Mb) in each pair of Chinese pangolin populations.

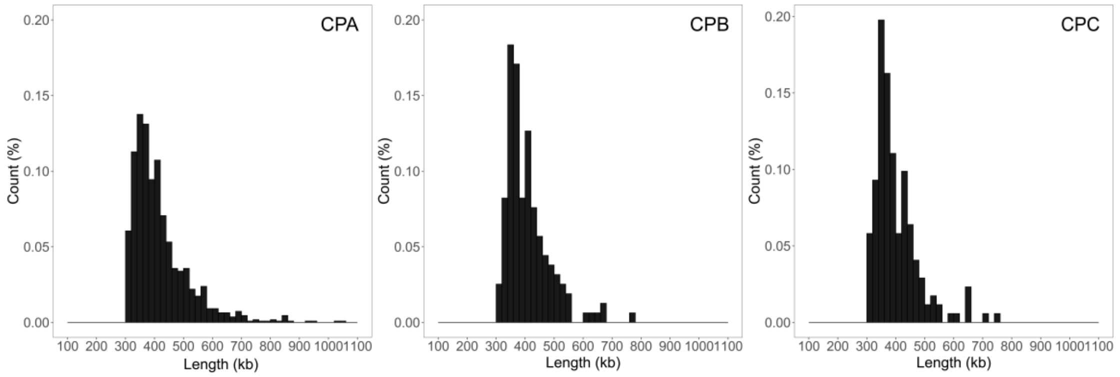

Supplementary Figure 7. The distribution of IBD segments in each Chinese pangolin population.

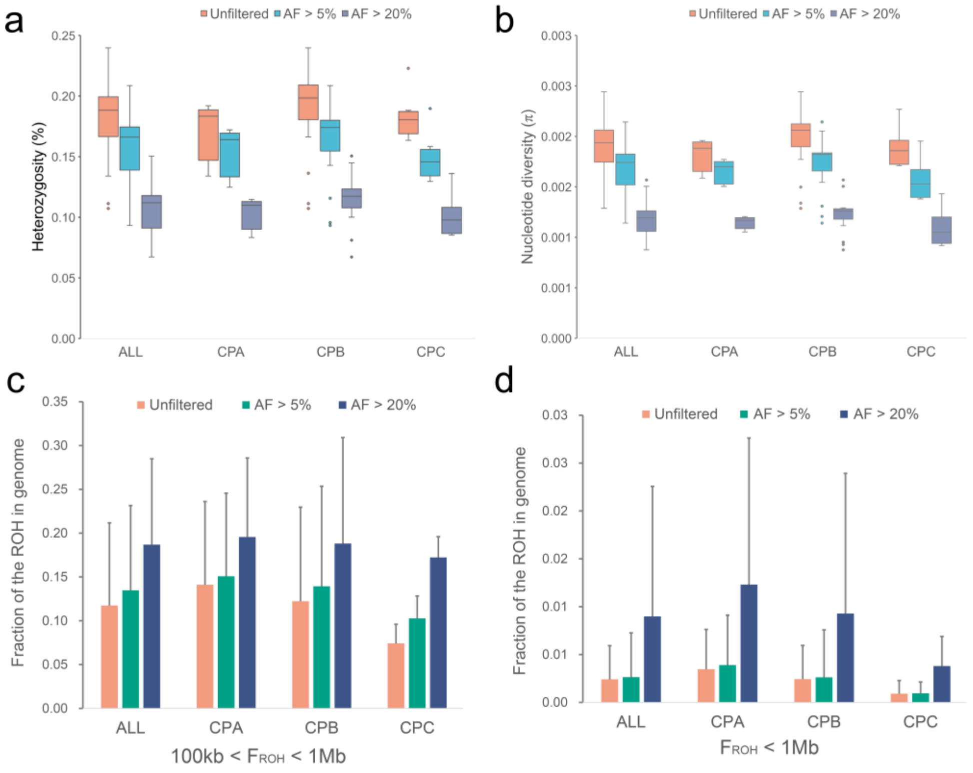

Supplementary Figure 8. Genetic diversity and run of homozygosity (ROH) in Chinese pangolin populations. (**a**) Heterozygosity of samples in each population. (**b**) Nucleotide diversity of samples in each population. (**c**) Fraction of ROHs (100 kb < F_{ROH} < 1 Mb) in the genome of each population. (**d**) Fraction of ROHs (F_{ROH} > 1 Mb) in the genome of each population. The standard error is presented by error bars.

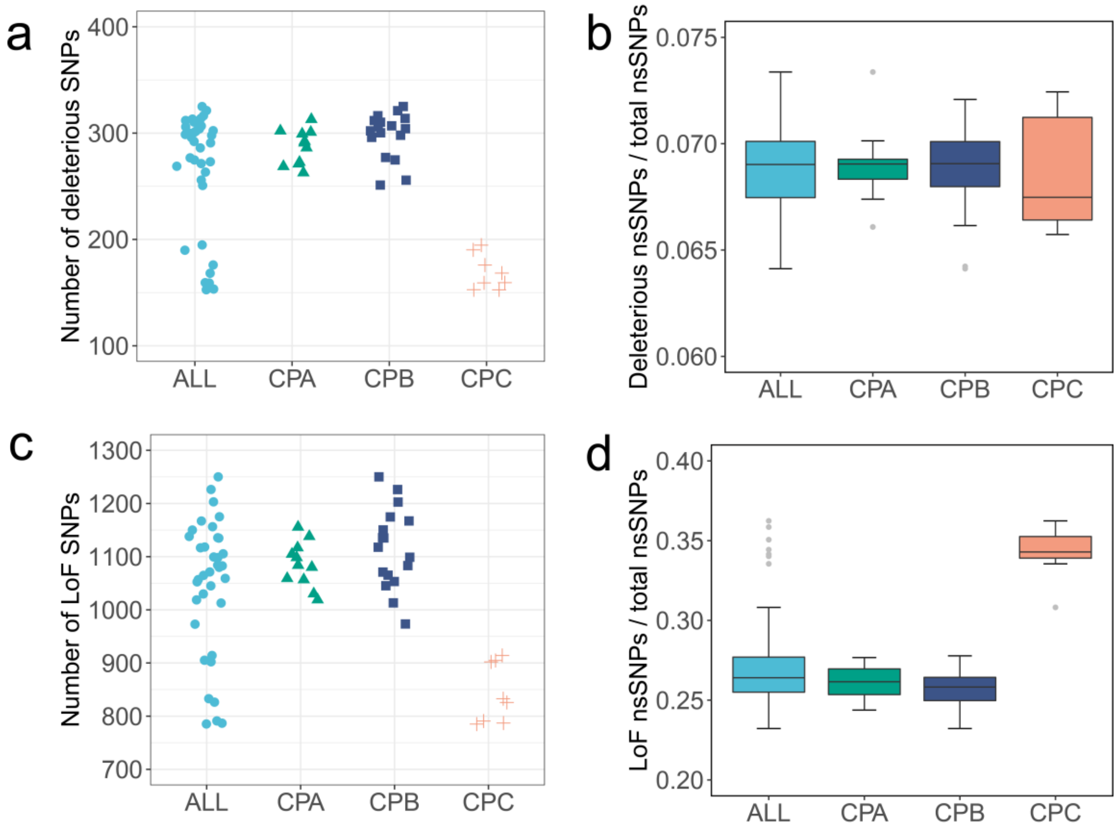

Supplementary Figure 9. The number and ratio of deleterious mutations and Loss of Function (LoF) variants in Chinese pangolin populations. (**a**) Number of deleterious mutations with Grantham Score (GS) ≥ 150 in each population. (**b**) The ratio of deleterious nonsynonymous SNPs (nsSNPs) to total nsSNPs in each population. (**c**) The number of Loss of Function (LoF) mutations in each population. (**d**) The ratio of LoF nsSNPs to total nsSNPs in each population. Error bars show range of values within 1.5 times the interquartile range. ALL: n = 36. CPA: n = 11. CPB: n = 17. CPC: n = 8.

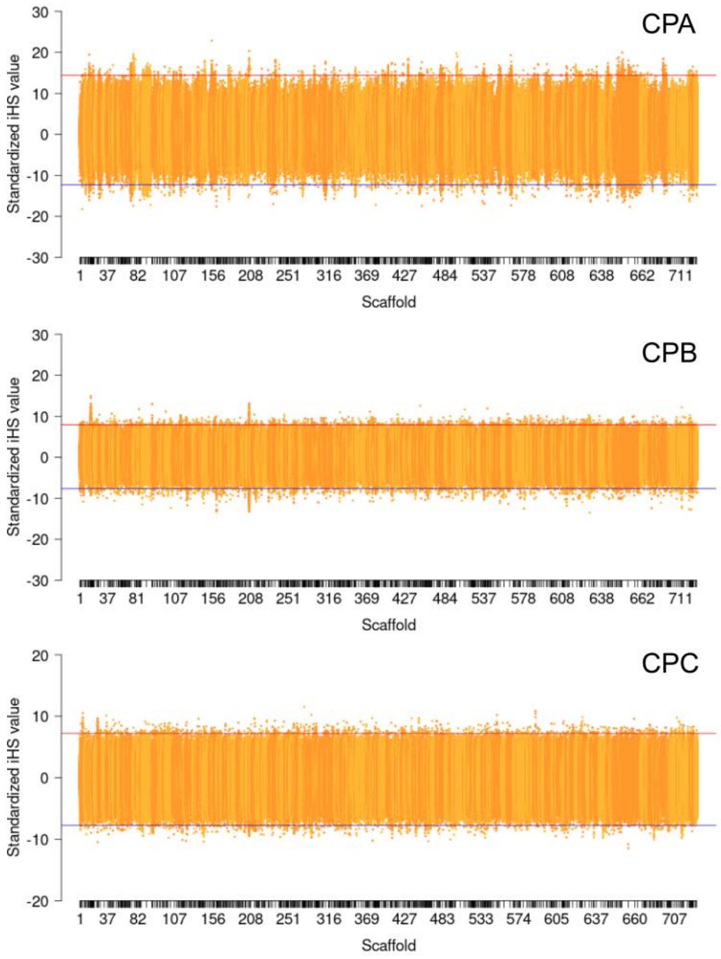

Supplementary Figure 10. Mutations under positive selection identified with the iHS method in three populations of Chinese pangolin. Red and black lines represent the 99.9% and 0.1% of standardized iHS values in each population. Standardized iHS values are plotted in logarithmic scale.

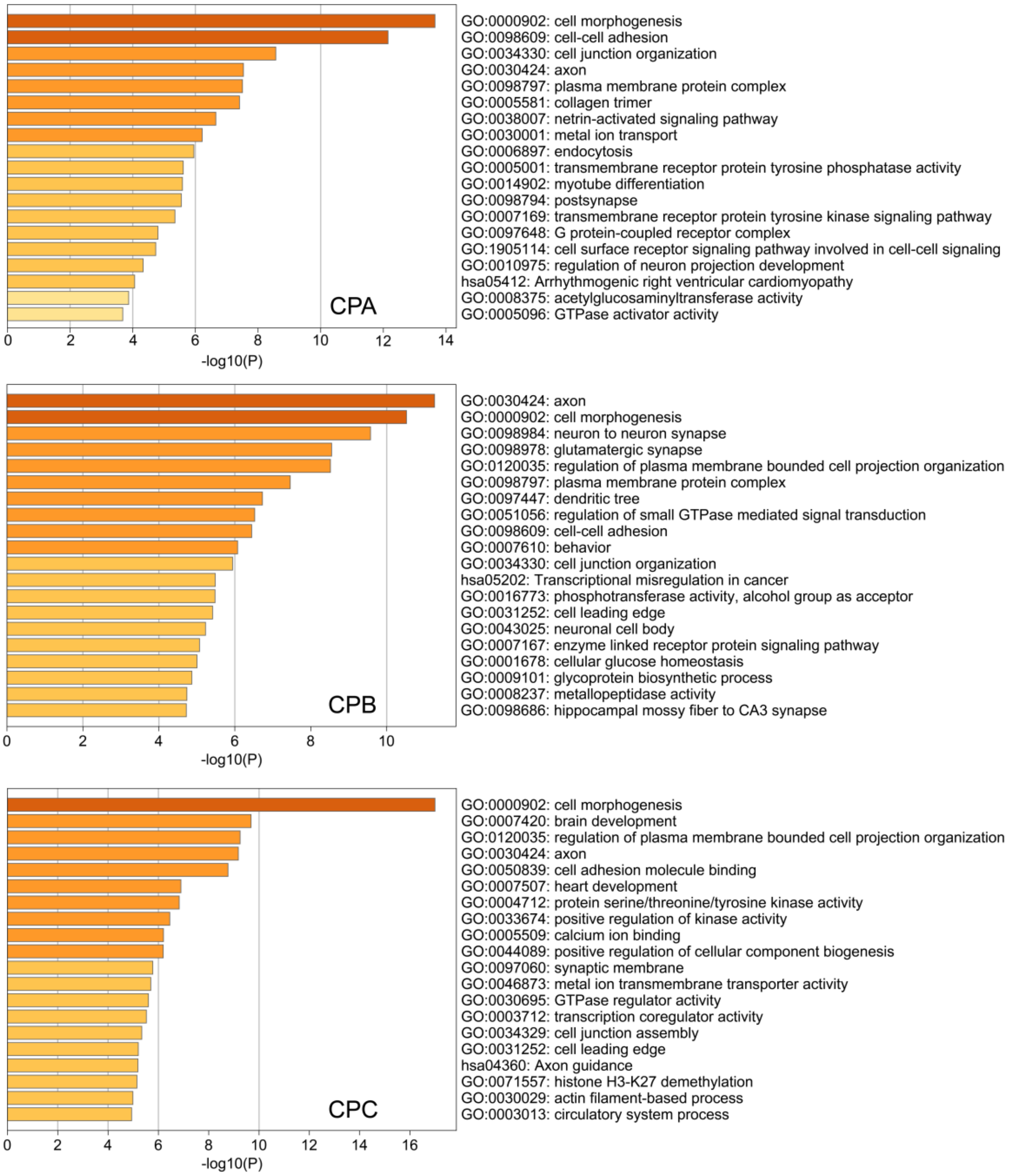

Supplementary Figure 11. Enrichment of the GO and KEGG pathway under positive selection identified by the iHS method in three Chinese pangolin populations. The top 20 clusters with their representative enriched terms with an adjusted *P*-value < 0.05 are displayed. Each bar is colored based on *P*-values. Log10(P) is the *P*-value in log base 10.

Supplementary Table 1. Samples and sequencing data of 37 Chinese pangolin individuals used in this study.

	Total	CPA	CPB	CPC
**Sample Size**	37	11	18	8
**Bases (Gb)**	81.33±35.33	40.99±4.37	94.53±31.83	108.03±10.95
**Depth (X)**	29.53±10.82	17.08±1.82	33.54±9.59	37.64±3.82
**Coverage**	0.95±0.01	0.95±0.00	0.95±0.01	0.95±0.00
**SNPs (Unfiltered)**	35,023,399	19,045,653	25,968,784	15,523,320
**SNPs (AF > 5%)**	20,310,861	16,009,692	17,887,160	10,870,731
**SNPs (AF > 20%)**	11,590,293	11,348,747	11,536,567	7,572,978
**InDels**	4,897,005	2,567,633	3,652,348	2,125,379
**Exon**	381,636	181,577	285,081	146,069
**Synonymous**	85,076	139,828	67,832	183,099
**Nonsynonymous**	81,069	123,870	65,966	170,852
**3'UTR**	295,640	143,537	220,401	113,205
**5'UTR**	91,953	46,582	67,190	36,413
**Intron**	12,809,925	6,881,876	9,418,940	5,563,082
**Intergenic**	19,487,686	10,718,113	14,519,412	8,785,006
**Splicing**	1,696	847	1,242	691
**non-coding RNA**	1,398,780	768,262	1,041,551	635,776

Supplementary Table 2. Sample information of 22 Chinese pangolin individuals downloaded from NCBI.

Sample ID	Previous ID*	NCBI SRR ID
MP-PRE-2	MP10 [A]	SRR9018583
MP-PRE-3	MP11 [A]	SRR9018584
MP-PRE-4	MP03 [A]	SRR9018585
MP-PRE-5	MP04 [A]	SRR9018590
MP-PRE-6	MP01 [A]	SRR9018591
MP-PRE-7	MP02 [A]	SRR9018592
MP-PRE-8	MP08 [A]	SRR9018593
MP-PRE-9	MP09 [A]	SRR9018594
MP-PRE-10	MP05 [A]	SRR9018595
MP-PRE-11	MP06 [A]	SRR9018596
MP-PRE-12	MP21 [B]	SRR9018600
MP-PRE-13	MP22 [B]	SRR9018601
MP-PRE-14	MP14 [A]	SRR9018602
MP-PRE-15	MP15 [B]	SRR9018603
MP-PRE-16	MP12 [A]	SRR9018604
MP-PRE-17	MP13 [A]	SRR9018605
MP-PRE-18	MP18 [B]	SRR9018606
MP-PRE-19	MP19 [B]	SRR9018607
MP-PRE-20	MP16 [B]	SRR9018608
MP-PRE-21	MP17 [B]	SRR9018609
MP-PRE-22	MP23 [B]	SRR9018653
Taiwan	MP07 [A]	SRR$^+$

* represents sample IDs were used in the Hu *et al* 's study [1]. [A] and [B] represents samples from MPA and MPB in the Hu *et al* 's study, respectively. SRR$^+$ represents sequence IDs of SRR770301, SRR770303, SRR770305, SRR770306, SRR770309, SRR770310, SRR770312, SRR770314, SRR770316, SRR770321, SRR770329, SRR770335, SRR770337, SRR770338, SRR770339, SRR770342, SRR770349, SRR770350, SRR770351, SRR770352 in the Choo *et al* 's study.[2]

Supplementary Table 3. Sample information of 72 Malayan pangolin individuals downloaded from NCBI.

Sample ID	Previous ID*	NCBI SRR ID	Sample ID	Previous ID*	NCBI SRR ID
MJ-PRE-1	MJ67	SRR9018586	MJ-PRE-37	MJ28	SRR9018639
MJ-PRE-2	MJ66	SRR9018587	MJ-PRE-38	MJ36	SRR9018640
MJ-PRE-3	MJ70	SRR9018588	MJ-PRE-39	MJ37	SRR9018641
MJ-PRE-4	MJ69	SRR9018589	MJ-PRE-40	MJ34	SRR9018642
MJ-PRE-5	MJ73	SRR9018597	MJ-PRE-41	MJ35	SRR9018643
MJ-PRE-6	MJ71	SRR9018598	MJ-PRE-42	MJ32	SRR9018644
MJ-PRE-7	MJ72	SRR9018599	MJ-PRE-43	MJ33	SRR9018645
MJ-PRE-8	MJ49	SRR9018610	MJ-PRE-44	MJ30	SRR9018646
MJ-PRE-9	MJ48	SRR9018611	MJ-PRE-45	MJ31	SRR9018647
MJ-PRE-10	MJ47	SRR9018612	MJ-PRE-46	MJ38	SRR9018648
MJ-PRE-11	MJ46	SRR9018613	MJ-PRE-47	MJ39	SRR9018649
MJ-PRE-12	MJ45	SRR9018614	MJ-PRE-48	MJ03	SRR9018650
MJ-PRE-13	MJ44	SRR9018615	MJ-PRE-49	MJ02	SRR9018651
MJ-PRE-14	MJ43	SRR9018616	MJ-PRE-50	MJ01	SRR9018652
MJ-PRE-15	MJ42	SRR9018617	MJ-PRE-51	MJ07	SRR9018654
MJ-PRE-16	MJ41	SRR9018618	MJ-PRE-52	MJ06	SRR9018655
MJ-PRE-17	MJ40	SRR9018619	MJ-PRE-53	MJ05	SRR9018656
MJ-PRE-18	MJ58	SRR9018620	MJ-PRE-54	MJ04	SRR9018657
MJ-PRE-19	MJ59	SRR9018621	MJ-PRE-55	MJ09	SRR9018658
MJ-PRE-20	MJ50	SRR9018622	MJ-PRE-56	MJ08	SRR9018659
MJ-PRE-21	MJ51	SRR9018623	MJ-PRE-57	MJ18	SRR9018660
MJ-PRE-22	MJ52	SRR9018624	MJ-PRE-58	MJ19	SRR9018661
MJ-PRE-23	MJ53	SRR9018625	MJ-PRE-59	MJ14	SRR9018662
MJ-PRE-24	MJ54	SRR9018626	MJ-PRE-60	MJ15	SRR9018663
MJ-PRE-25	MJ55	SRR9018627	MJ-PRE-61	MJ16	SRR9018664
MJ-PRE-26	MJ56	SRR9018628	MJ-PRE-62	MJ17	SRR9018665
MJ-PRE-27	MJ57	SRR9018629	MJ-PRE-63	MJ10	SRR9018666
MJ-PRE-28	MJ25	SRR9018630	MJ-PRE-64	MJ11	SRR9018667
MJ-PRE-29	MJ24	SRR9018631	MJ-PRE-65	MJ12	SRR9018668
MJ-PRE-30	MJ27	SRR9018632	MJ-PRE-66	MJ13	SRR9018669
MJ-PRE-31	MJ26	SRR9018633	MJ-PRE-67	MJ61	SRR9018670
MJ-PRE-32	MJ21	SRR9018634	MJ-PRE-68	MJ60	SRR9018671
MJ-PRE-33	MJ20	SRR9018635	MJ-PRE-69	MJ63	SRR9018672
MJ-PRE-34	MJ23	SRR9018636	MJ-PRE-70	MJ62	SRR9018673
MJ-PRE-35	MJ22	SRR9018637	MJ-PRE-71	MJ65	SRR9018674
MJ-PRE-36	MJ29	SRR9018638	MJ-PRE-72	MJ64	SRR9018675

*Sample IDs in the published study [1].

Supplementary Table 4. The *P*-values of the ration of mutational load in each pair of Chinese pangolins.

Type of mutational load	CPA v.s. CPB	CPA v.s. CPC	CPB v.s. CPC
Loss of function (LoF) mutations	0.235	3.44×10^{-07}	8.123×10^{-06}
Missense mutations	0.089	1.900×10^{-13}	3.663×10^{-12}
Deleterious missense mutations	0.030	1.205×10^{-11}	5.711×10^{-13}

Supplementary Table 5. GO and KEGG enrichment analyses of 2229 genes affected by LoF mutations across 37 Chinese pangolins.

Term	Description	Log10(P)	Log10(q)	Genes in list	Total genes
GO:0007017	microtubule-based process	-16.50	-12.30	122	763
GO:0048232	male gamete generation	-12.42	-8.63	93	588
GO:0007601	visual perception	-9.05	-5.97	43	217
GO:0030031	cell projection assembly	-12.35	-8.63	65	415
GO:0031589	cell-substrate adhesion	-8.33	-5.42	38	188
KEGG: hsa04974	Protein digestion and absorption	-5.52	-2.97	22	103
KEGG: hsa04910	Insulin signaling pathway	-5.39	-2.85	26	137
GO:0016311	dephosphorylation	-5.37	-2.85	45	309
GO:1901605	alpha-amino acid metabolic process	-5.06	-2.59	32	195
GO:0044262	cellular carbohydrate metabolic process	-4.99	-2.55	28	161
GO:0018108	peptidyl-tyrosine phosphorylation	-5.17	-2.68	26	146
GO:0030198	extracellular matrix organization	-5.07	-2.59	39	266
KEGG: hsa04510	Focal adhesion	-4.78	-2.40	32	201
GO:0007015	actin filament organization	-4.77	-2.40	39	267
KEGG: hsa04020	Calcium signaling pathway	-4.71	-2.36	36	240
GO:0018209	peptidyl-serine modification	-5.06	-2.59	32	206
KEGG: hsa01240	Biosynthesis of cofactors	-4.50	-2.18	26	153
GO:0006936	muscle contraction	-4.47	-2.16	34	227
GO:0006739	NADP metabolic process	-4.29	-2.00	10	32
KEGG: hsa04146	Peroxisome	-4.24	-1.96	17	82

"Log10(P)" is the *P* -value in log base 10. "Log10(q)" is the multi-test adjusted *P* -value in log base 10.

Supplementary Table 6. GO and KEGG enrichment analyses of 1044 genes affected by LoF mutations in the CPA population.

Term	Description	Log10(P)	Log10(q)	Genes in list	Total genes
KEGG: hsa04974	Protein digestion and absorption	-6.48	-2.28	16	103
GO:0048232	male gamete generation	-6.13	-2.23	44	588
GO:0031589	cell-substrate adhesion	-5.81	-2.09	21	188
GO:0045229	external encapsulating structure organization	-4.83	-1.48	24	269

"Log10(P)" is the *P* -value in log base 10. "Log10(q)" is the multi-test adjusted *P* -value in log base 10.

Supplementary Table 7. GO and KEGG enrichment analyses of 1,574 genes affected by LoF mutations in the CPB population.

Term	Description	Log10(P)	Log10(q)	Genes in list	Total genes
GO:0048232	male gamete generation	-8.86	-5.05	66	588
GO:0050953	sensory perception of light stimulus	-7.78	-4.43	33	221
KEGG: hsa04974	Protein digestion and absorption	-6.04	-3.05	19	103
GO:0006820	anion transport	-5.34	-2.59	46	447
GO:0045229	external encapsulating structure organization	-5.15	-2.43	32	269
KEGG: hsa04510	Focal adhesion	-4.95	-2.27	26	201
GO:0044782	cilium organization	-5.07	-2.37	37	343
KEGG: hsa04146	Peroxisome	-4.85	-2.22	15	82
GO:0032940	secretion by cell	-4.81	-2.22	39	372
KEGG: hsa04910	Insulin signaling pathway	-4.19	-1.73	19	137
GO:1902600	proton transmembrane transport	-4.04	-1.64	18	129
GO:0007605	sensory perception of sound	-3.90	-1.53	20	156
GO:0044262	cellular carbohydrate metabolic process	-3.71	-1.38	20	161
GO:0009750	response to fructose	-3.69	-1.37	4	7
GO:0097722	sperm motility	-8.82	-5.05	15	103
GO:0035524	proline transmembrane transport	-4.85	-2.22	4	8

"Log10(P)" is the *P* -value in log base 10. "Log10(q)" is the multi-test adjusted *P* -value in log base 10.

Supplementary Table 8. GO and KEGG enrichment analyses of 962 genes affected by LoF mutations in the CPC population.

Term	Description	Log10(P)	Log10(q)	Genes in list	Total genes
GO:0030036	actin cytoskeleton organization	-7.72	-3.92	43	540
GO:0007017	microtubule-based process	-6.97	-3.87	52	763
KEGG: hsa04974	Protein digestion and absorption	-5.46	-2.56	14	103
GO:1901605	alpha-amino acid metabolic process	-4.42	-1.67	18	195
GO:0006897	endocytosis	-4.19	-1.51	32	489
GO:0010520	regulation of reciprocal meiotic recombination	-3.94	-1.33	3	4

"Log10(P)" is the *P*-value in log base 10. "Log10(q)" is the multi-test adjusted *P* -value in log base 10.

Supplementary reference

1. Hu, J.-Y., Hao, Z.-Q., Frantz, L., Wu, S.-F., Chen, W., Jiang, Y.-F., Wu, H., Kuang, W.-M., Li, H., and Zhang, Y.-P. (2020). Genomic consequences of population decline in critically endangered pangolins and their demographic histories. National Science Review *7*, 798-814.
2. Choo, S.W., Rayko, M., Tan, T.K., Hari, R., Komissarov, A., Wee, W.Y., Yurchenko, A.A., Kliver, S., Tamazian, G., and Antunes, A. (2016). Pangolin genomes and the evolution of mammalian scales and immunity. Genome research *26*, 1312-1322.
