# Supplementary material for: Whole-genome resequencing of Chinese pangolins reveals a population structure and provides insights into their conservation
Source: Commun Biol. 2022 Aug 25;5:821. doi: 10.1038/s42003-022-03757-3 (PMC9411537; doi:10.1038/s42003-022-03757-3)
Supplement: Supplementary file 2 — Description of Additional Supplementary Files [file 42003_2022_3757_MOESM2_ESM.pdf]

# Description of Additional Supplementary Files

**File name:** Supplementary Data 1
**Description:** The source data behind the figures in the main manuscript.
